# (*E*)-Methyl 3-(3,4-dimeth­oxy­phen­yl)-2-[(1,3-dioxoisoindolin-2-yl)meth­yl]acrylate

**DOI:** 10.1107/S1600536812010975

**Published:** 2012-03-17

**Authors:** D. Kannan, M. Bakthadoss, D. Lakshmanan, S. Murugavel

**Affiliations:** a Department of Organic Chemistry, University of Madras, Maraimalai Campus, Chennai 600 025, India; b Department of Physics, C. Abdul Hakeem College of Engineering & Technology, Melvisharam, Vellore 632 509, India; c Department of Physics, Thanthai Periyar Government Institute of Technology, Vellore 632 002, India

## Abstract

In the title compound, C_21_H_19_NO_6_, the isoindole ring system is essentially planar [maximum deviation = 0.019 (2) Å for the N atom] and is oriented at a dihedral angle of 51.3 (1)° with respect to the benzene ring. The two meth­oxy groups are almost coplanar with the attached benzene ring [C—O—C—C = 3.7 (4) and 4.3 (4)°]. The mol­ecular conformation is stabilized by an intra­molecular C—H⋯O hydrogen bond, which generates an *S*(9) ring motif. In the crystal, mol­ecules are linked through bifurcated C—H⋯(O,O) hydrogen bonds having *R*_1_^2^(5) ring motifs, forming chains along the *b*-axis direction. The crystal packing is further stabilzed by π–π inter­actions [centriod–centroid distance = 3.463 (1) Å].

## Related literature

For background to the applications of isoindolins, see: Pendrak *et al.* (1994 ▶); De Clerck (1995 ▶); Stowers (1996 ▶); Heaney & Shuhaibar (1995 ▶). For related structures, see: Liu *et al.* (2004 ▶); Liang & Li (2006 ▶). For hydrogen-bond motifs, see: Bernstein *et al.* (1995 ▶).
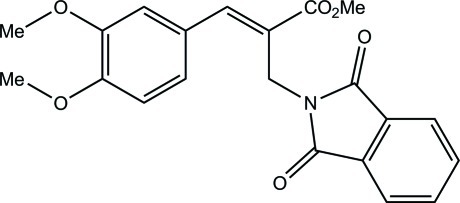


## Experimental

### 

#### Crystal data

C_21_H_19_NO_6_
*M* *_r_* = 381.37
Monoclinic, 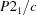

*a* = 15.0613 (8) Å
*b* = 7.6334 (4) Å
*c* = 16.6354 (8) Å
β = 93.522 (2)°
*V* = 1908.94 (17) Å^3^
*Z* = 4
Mo *K*α radiation
μ = 0.10 mm^−1^
*T* = 293 K
0.25 × 0.23 × 0.17 mm

#### Data collection

Bruker APEXII CCD diffractometer
Absorption correction: multi-scan (*SADABS*; Sheldrick, 1996 ▶) *T*_min_ = 0.976, *T*_max_ = 0.983
21209 measured reflections
5063 independent reflections
3241 reflections with *I* > 2σ(*I*)
*R*_int_ = 0.036

#### Refinement

*R*[*F* ^2^ > 2σ(*F* ^2^)] = 0.055
*wR*(*F* ^2^) = 0.197
*S* = 1.06
5063 reflections
257 parameters
H-atom parameters constrained
Δρ_max_ = 0.26 e Å^−3^
Δρ_min_ = −0.17 e Å^−3^

### 

Data collection: *APEX2* (Bruker, 2004 ▶); cell refinement: *APEX2* and *SAINT* (Bruker, 2004 ▶); data reduction: *SAINT* and *XPREP* (Bruker, 2004 ▶); program(s) used to solve structure: *SHELXS97* (Sheldrick, 2008 ▶); program(s) used to refine structure: *SHELXL97* (Sheldrick, 2008 ▶); molecular graphics: *ORTEP-3* (Farrugia, 1997 ▶); software used to prepare material for publication: *SHELXL97* and *PLATON* (Spek, 2009 ▶).

## Supplementary Material

Crystal structure: contains datablock(s) global, I. DOI: 10.1107/S1600536812010975/bt5842sup1.cif

Structure factors: contains datablock(s) I. DOI: 10.1107/S1600536812010975/bt5842Isup2.hkl

Supplementary material file. DOI: 10.1107/S1600536812010975/bt5842Isup3.cml

Additional supplementary materials:  crystallographic information; 3D view; checkCIF report

## Figures and Tables

**Table 1:** Hydrogen-bond geometry (Å, °)

*D*—H⋯*A*	*D*—H	H⋯*A*	*D*⋯*A*	*D*—H⋯*A*
C15—H15⋯O1	0.93	2.55	3.440 (3)	160
C4—H4⋯O5^i^	0.93	2.50	3.354 (3)	153
C4—H4⋯O6^i^	0.93	2.58	3.320 (4)	137

Symmetry code: (i) 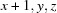.

**Fig. 1.**
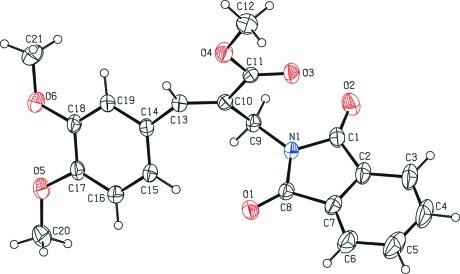
The molecular structure of the title compound with the atom numbering scheme. Displacement ellipsoids are drawn at the 30% probability level. H atoms are presented as a small cycles of arbitrary radius.

**Fig. 2.**
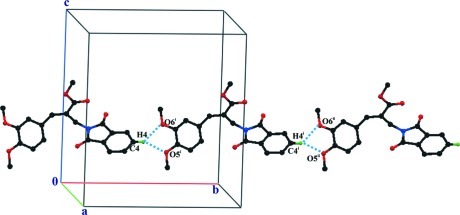
Part of the crystal structure of (I) showing bifurcated C—H···O hydrogen bonds (dotted lines) generating R12(5) ring motifs, forming one dimensional extended chains along the b axis. Only the H atoms involved the hydrogen bonds are shown. [Symmetry codes:(i)1 + x, y, z; (ii)2 + x, y, z].

**Fig. 3.**
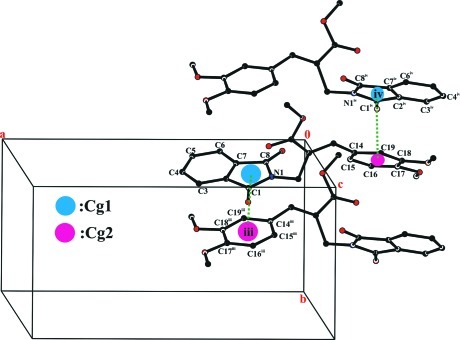
A view of the π—π interactions (dotted lines) in the crystal structure of the title compound. Cg1 and Cg2 denotes centroids of the N1/C1/C2/C7/C8 indole ring and C14–C19 benzene ring, respectively. [Symmetry codes: (iii)-x, 1/2 + y, 1/2 - z; (iv)-x, -1/2 + y, 1/2 - z].

## supplementary crystallographic information

### Comment

Isoindolinones and their derivatives have been investigated widely due to their physiological and chemotherapeutic properties. Many compounds containing the isoindolinone skeleton have shown antiviral, antileukemic, antiinflammatory, antipsychotic and antiulcer properties (Pendrak *et al.*, 1994; De Clerck, 1995). Isoindolinones are useful for the synthesis of various drugs and naturally occurring compounds (Stowers, 1996; Heaney & Shuhaibar, 1995). In view of this biological importance, the crystal structure of the title compound has been determined and the results are presented here.

Fig. 1. shows a displacement ellipsoid plot of (I), with the atom numbering scheme. The isoindole ring system is essentially planar [maximum deviation = 0.019 (2) Å for the N1 atom] and is oriented at a dihedral angle of 51.3 (1)° with respect to the benzene ring. The methyl acrylate (O3/O4/C10–C14) plane forms dihedral angles of 83.2 (1)° and 43.7 (1)°, respectively, with the isoindole and benzene rings. The two methoxy groups at C18 and C17 are almost coplanar with the attached benzene ring as evidenced by torsion angles of C21–O6–C18–C19 = 3.7 (4) and C20–O5–C17–C16 = 4.3 (4)°, respectively. The sum of bond angles around N1 (359.9°) indicates that N1 is in *sp*^2^ hybridization. The keto atoms O1 and O2 deviate by 0.034 (2) and -0.004 (2) Å, respectively, from the isoindole ring. The geometric parameters of the title molecule agrees well with those reported for similar structures (Liu *et al.*, 2004, Liang & Li 2006).

The molecular structure is stabilized by C15—H15···O1 intramolecular hydrogen bond, forming S(9) ring motif (Bernstein *et al.*, 1995) (Table 1). In the crystal, the molecules are linked by intermolecular C4—H4···O5^i^ and C4—H4···O6^i^ hydrogen bonds (Table 1; Symmetry code: (i) = *1 + x, y, z)*) generating a bifurcated *R_1_^2^*(5) ring motif, resulting in an extended one dimensional chains along the *b* axis (Fig. 2). The crystal packing is further stabilized by π—π interactions with centroid—centroid distances: *Cg*1—*Cg*2^iii^ = 3.463 (1) Å and *Cg*2—*Cg*1^iv^ = 3.463 (1) Å (Fig. 3; *Cg*1 and *Cg*2 are the centroids of N1/C1/C2/C7/C8 indole ring and C14–C19 benzene ring, respectively, symmetry code as in Fig. 3).

### Experimental

A solution of 2,3-dihydro-1*H*-isoindole-1,3-dione (1 mmol, 0.147 g) and potassiumcarbonate (1.5 mmol, 0.207 g) in acetonitrile as solvent was stirred for 15 minutes at room temperature. To this solution, methyl (2*Z*)-2-(bromomethyl)-3-(3,4-dimethoxyphenyl)prop-2-enoate (1 mmol, 0.315 g) was added till the addition is complete. After the completion of the reaction as indicated by TLC, acetonitrile solvent was evaporated. Ethylacetate (15 ml) and water (15 ml) were added to the crude mass. The organic layer was dried over anhydrous sodium sulfate. Removal of solvent led to the crude product, which was purified through pad of silica gel (100–200 mesh) using ethylacetate and hexanes (1:9) as solvents. The pure title compound was obtained as a colorless solid (0.375 g, 98% yield). Recrystallization was carried out using ethylacetate as solvent.

### Refinement

H atoms were positioned geometrically, with C—H = 0.93–0.98 Å and constrained to ride on their parent atom, with *U*_iso_(H)=1.5*U*_eq_ for methyl H atoms and 1.2*U*_eq_(C) for other H atoms.

### Crystal data

**Table:**

C_21_H_19_NO_6_	*F*(000) = 800
*M**_r_* = 381.37	*D*_x_ = 1.327 Mg m^−^^3^
Monoclinic, *P*2_1_/*c*	Mo *K*α radiation, λ = 0.71073 Å
Hall symbol: -P 2ybc	Cell parameters from 5078 reflections
*a* = 15.0613 (8) Å	θ = 2.7–29.0°
*b* = 7.6334 (4) Å	µ = 0.10 mm^−^^1^
*c* = 16.6354 (8) Å	*T* = 293 K
β = 93.522 (2)°	Block, colourless
*V* = 1908.94 (17) Å^3^	0.25 × 0.23 × 0.17 mm
*Z* = 4	

### Data collection

**Table:**

Bruker APEXII CCD diffractometer	5063 independent reflections
Radiation source: fine-focus sealed tube	3241 reflections with *I* > 2σ(*I*)
Graphite monochromator	*R*_int_ = 0.036
Detector resolution: 10.0 pixels mm^-1^	θ_max_ = 29.0°, θ_min_ = 2.7°
ω scans	*h* = −20→20
Absorption correction: multi-scan (*SADABS*; Sheldrick, 1996)	*k* = −10→9
*T*_min_ = 0.976, *T*_max_ = 0.983	*l* = −14→22
21209 measured reflections	

### Refinement

**Table:**

Refinement on *F*^2^	Secondary atom site location: difference Fourier map
Least-squares matrix: full	Hydrogen site location: inferred from neighbouring sites
*R*[*F*^2^ > 2σ(*F*^2^)] = 0.055	H-atom parameters constrained
*wR*(*F*^2^) = 0.197	*w* = 1/[σ^2^(*F*_o_^2^) + (0.0874*P*)^2^ + 0.8631*P*] where *P* = (*F*_o_^2^ + 2*F*_c_^2^)/3
*S* = 1.06	(Δ/σ)_max_ < 0.001
5063 reflections	Δρ_max_ = 0.26 e Å^−^^3^
257 parameters	Δρ_min_ = −0.17 e Å^−^^3^
0 restraints	Extinction correction: *SHELXL97* (Sheldrick, 2008), Fc^*^=kFc[1+0.001xFc^2^λ^3^/sin(2θ)]^-1/4^
Primary atom site location: structure-invariant direct methods	Extinction coefficient: 0.010 (2)

### Special details

**Table:**

Geometry. All e.s.d.'s (except the e.s.d. in the dihedral angle between two l.s. planes) are estimated using the full covariance matrix. The cell e.s.d.'s are taken into account individually in the estimation of e.s.d.'s in distances, angles and torsion angles; correlations between e.s.d.'s in cell parameters are only used when they are defined by crystal symmetry. An approximate (isotropic) treatment of cell e.s.d.'s is used for estimating e.s.d.'s involving l.s. planes.
Refinement. Refinement of *F*^2^ against ALL reflections. The weighted *R*-factor *wR* and goodness of fit *S* are based on *F*^2^, conventional *R*-factors *R* are based on *F*, with *F* set to zero for negative *F*^2^. The threshold expression of *F*^2^ > σ(*F*^2^) is used only for calculating *R*-factors(gt) *etc*. and is not relevant to the choice of reflections for refinement. *R*-factors based on *F*^2^ are statistically about twice as large as those based on *F*, and *R*- factors based on ALL data will be even larger.

### Fractional atomic coordinates and isotropic or equivalent isotropic displacement parameters (Å^2^)

**Table:**

	*x*	*y*	*z*	*U*_iso_*/*U*_eq_	
N1	0.14320 (11)	0.1401 (2)	0.33185 (10)	0.0424 (4)	
O1	0.09640 (11)	0.0065 (3)	0.21318 (10)	0.0573 (5)	
O3	0.16679 (10)	−0.1006 (2)	0.45930 (10)	0.0548 (4)	
C14	−0.13047 (13)	−0.0184 (3)	0.32900 (12)	0.0406 (5)	
O5	−0.35924 (10)	0.1490 (3)	0.20022 (11)	0.0632 (5)	
O2	0.23094 (13)	0.2752 (3)	0.43312 (11)	0.0670 (5)	
O6	−0.37165 (11)	0.0404 (3)	0.34609 (11)	0.0733 (6)	
C11	0.09073 (13)	−0.1416 (3)	0.44408 (11)	0.0393 (5)	
C13	−0.05573 (13)	−0.0948 (3)	0.37754 (12)	0.0424 (5)	
H13	−0.0679	−0.2017	0.4013	0.051*	
O4	0.05602 (11)	−0.2889 (2)	0.47147 (10)	0.0562 (4)	
C16	−0.19917 (14)	0.1059 (3)	0.20806 (13)	0.0464 (5)	
H16	−0.1940	0.1488	0.1562	0.056*	
C15	−0.12410 (13)	0.0491 (3)	0.25270 (13)	0.0445 (5)	
H15	−0.0688	0.0566	0.2310	0.053*	
C10	0.02695 (13)	−0.0361 (3)	0.39320 (11)	0.0382 (4)	
C8	0.15518 (13)	0.0666 (3)	0.25730 (13)	0.0434 (5)	
C17	−0.28144 (13)	0.0993 (3)	0.23975 (14)	0.0456 (5)	
C9	0.05918 (13)	0.1426 (3)	0.37010 (13)	0.0415 (5)	
H9A	0.0145	0.1966	0.3337	0.050*	
H9B	0.0656	0.2148	0.4181	0.050*	
C19	−0.21432 (14)	−0.0266 (3)	0.36066 (13)	0.0460 (5)	
H19	−0.2199	−0.0754	0.4113	0.055*	
C18	−0.28843 (13)	0.0364 (3)	0.31812 (13)	0.0472 (5)	
C1	0.22284 (15)	0.2025 (3)	0.36886 (15)	0.0493 (5)	
C7	0.25214 (15)	0.0779 (3)	0.24589 (15)	0.0515 (6)	
C2	0.29162 (14)	0.1602 (3)	0.31177 (16)	0.0538 (6)	
C12	0.11416 (19)	−0.3925 (4)	0.52426 (17)	0.0632 (7)	
H12A	0.1582	−0.4481	0.4936	0.095*	
H12B	0.0801	−0.4803	0.5499	0.095*	
H12C	0.1430	−0.3183	0.5645	0.095*	
C20	−0.35659 (18)	0.2005 (5)	0.11918 (18)	0.0733 (8)	
H20A	−0.3193	0.3019	0.1157	0.110*	
H20B	−0.4156	0.2281	0.0980	0.110*	
H20C	−0.3330	0.1066	0.0885	0.110*	
C3	0.38247 (17)	0.1899 (4)	0.3186 (2)	0.0751 (9)	
H3	0.4095	0.2460	0.3633	0.090*	
C6	0.29985 (19)	0.0199 (4)	0.1836 (2)	0.0729 (8)	
H6	0.2727	−0.0361	0.1390	0.087*	
C21	−0.3808 (2)	−0.0149 (7)	0.42584 (19)	0.1007 (14)	
H21A	−0.3607	−0.1338	0.4318	0.151*	
H21B	−0.4421	−0.0078	0.4380	0.151*	
H21C	−0.3457	0.0591	0.4620	0.151*	
C4	0.43088 (19)	0.1328 (6)	0.2565 (3)	0.0924 (11)	
H4	0.4920	0.1512	0.2589	0.111*	
C5	0.3909 (2)	0.0494 (6)	0.1909 (3)	0.0950 (12)	
H5	0.4258	0.0114	0.1501	0.114*	

### Atomic displacement parameters (Å^2^)

**Table:**

	*U*^11^	*U*^22^	*U*^33^	*U*^12^	*U*^13^	*U*^23^
N1	0.0315 (8)	0.0505 (10)	0.0451 (9)	−0.0048 (7)	0.0017 (7)	0.0023 (8)
O1	0.0408 (8)	0.0798 (12)	0.0511 (9)	−0.0029 (8)	0.0002 (7)	−0.0105 (8)
O3	0.0353 (8)	0.0664 (11)	0.0615 (10)	−0.0024 (7)	−0.0063 (7)	0.0094 (8)
C14	0.0318 (9)	0.0448 (11)	0.0450 (11)	−0.0035 (8)	−0.0001 (8)	−0.0056 (9)
O5	0.0325 (8)	0.0895 (14)	0.0664 (11)	0.0066 (8)	−0.0053 (7)	0.0092 (10)
O2	0.0611 (11)	0.0705 (12)	0.0675 (11)	−0.0154 (9)	−0.0101 (9)	−0.0092 (10)
O6	0.0311 (8)	0.1258 (18)	0.0637 (11)	0.0017 (10)	0.0091 (7)	0.0027 (11)
C11	0.0340 (10)	0.0487 (12)	0.0357 (9)	0.0010 (8)	0.0059 (7)	−0.0045 (9)
C13	0.0365 (10)	0.0470 (12)	0.0437 (10)	−0.0024 (9)	0.0024 (8)	0.0000 (9)
O4	0.0451 (9)	0.0584 (10)	0.0644 (10)	−0.0024 (7)	−0.0021 (7)	0.0178 (8)
C16	0.0379 (11)	0.0550 (13)	0.0463 (11)	−0.0013 (9)	0.0018 (9)	0.0039 (10)
C15	0.0304 (9)	0.0537 (13)	0.0496 (11)	−0.0020 (9)	0.0038 (8)	−0.0007 (10)
C10	0.0329 (9)	0.0450 (11)	0.0370 (9)	0.0023 (8)	0.0043 (7)	−0.0043 (8)
C8	0.0333 (10)	0.0498 (12)	0.0473 (11)	0.0004 (9)	0.0036 (8)	0.0078 (9)
C17	0.0287 (10)	0.0536 (13)	0.0537 (12)	−0.0002 (9)	−0.0031 (8)	−0.0044 (10)
C9	0.0317 (10)	0.0455 (12)	0.0474 (11)	0.0005 (8)	0.0034 (8)	−0.0016 (9)
C19	0.0349 (10)	0.0601 (14)	0.0428 (11)	−0.0061 (9)	0.0017 (8)	−0.0036 (10)
C18	0.0281 (9)	0.0636 (14)	0.0503 (12)	−0.0037 (9)	0.0049 (8)	−0.0070 (10)
C1	0.0387 (11)	0.0500 (13)	0.0580 (13)	−0.0077 (9)	−0.0063 (9)	0.0083 (11)
C7	0.0374 (11)	0.0564 (14)	0.0613 (13)	0.0008 (10)	0.0086 (10)	0.0134 (11)
C2	0.0319 (11)	0.0551 (14)	0.0739 (16)	−0.0022 (10)	−0.0009 (10)	0.0179 (12)
C12	0.0630 (16)	0.0607 (16)	0.0651 (15)	0.0061 (12)	−0.0027 (12)	0.0180 (13)
C20	0.0498 (15)	0.096 (2)	0.0717 (17)	0.0044 (14)	−0.0136 (12)	0.0157 (16)
C3	0.0358 (13)	0.083 (2)	0.105 (2)	−0.0079 (13)	−0.0043 (14)	0.0220 (17)
C6	0.0498 (15)	0.091 (2)	0.0801 (18)	0.0045 (14)	0.0206 (13)	0.0042 (16)
C21	0.0527 (17)	0.184 (4)	0.0680 (18)	−0.005 (2)	0.0229 (14)	0.006 (2)
C4	0.0316 (13)	0.115 (3)	0.131 (3)	0.0001 (15)	0.0126 (16)	0.025 (2)
C5	0.0522 (17)	0.117 (3)	0.119 (3)	0.0103 (19)	0.0360 (19)	0.013 (2)

### Geometric parameters (Å, º)

**Table:**

N1—C8	1.383 (3)	C9—H9A	0.9700
N1—C1	1.398 (3)	C9—H9B	0.9700
N1—C9	1.451 (3)	C19—C18	1.371 (3)
O1—C8	1.205 (3)	C19—H19	0.9300
O3—C11	1.200 (2)	C1—C2	1.483 (4)
C14—C15	1.379 (3)	C7—C2	1.367 (4)
C14—C19	1.399 (3)	C7—C6	1.370 (4)
C14—C13	1.465 (3)	C2—C3	1.385 (3)
O5—C17	1.362 (3)	C12—H12A	0.9600
O5—C20	1.407 (3)	C12—H12B	0.9600
O2—C1	1.204 (3)	C12—H12C	0.9600
O6—C18	1.364 (3)	C20—H20A	0.9600
O6—C21	1.407 (4)	C20—H20B	0.9600
C11—O4	1.332 (3)	C20—H20C	0.9600
C11—C10	1.479 (3)	C3—C4	1.372 (5)
C13—C10	1.334 (3)	C3—H3	0.9300
C13—H13	0.9300	C6—C5	1.388 (4)
O4—C12	1.438 (3)	C6—H6	0.9300
C16—C17	1.377 (3)	C21—H21A	0.9600
C16—C15	1.383 (3)	C21—H21B	0.9600
C16—H16	0.9300	C21—H21C	0.9600
C15—H15	0.9300	C4—C5	1.370 (6)
C10—C9	1.505 (3)	C4—H4	0.9300
C8—C7	1.487 (3)	C5—H5	0.9300
C17—C18	1.399 (3)		
			
C8—N1—C1	112.17 (18)	C19—C18—C17	119.71 (19)
C8—N1—C9	124.33 (17)	O2—C1—N1	125.8 (2)
C1—N1—C9	123.38 (19)	O2—C1—C2	129.2 (2)
C15—C14—C19	118.60 (19)	N1—C1—C2	104.9 (2)
C15—C14—C13	124.16 (18)	C2—C7—C6	122.1 (2)
C19—C14—C13	117.08 (19)	C2—C7—C8	107.8 (2)
C17—O5—C20	117.79 (19)	C6—C7—C8	130.0 (3)
C18—O6—C21	117.4 (2)	C7—C2—C3	121.3 (3)
O3—C11—O4	122.55 (19)	C7—C2—C1	109.08 (19)
O3—C11—C10	123.8 (2)	C3—C2—C1	129.6 (3)
O4—C11—C10	113.62 (17)	O4—C12—H12A	109.5
C10—C13—C14	130.6 (2)	O4—C12—H12B	109.5
C10—C13—H13	114.7	H12A—C12—H12B	109.5
C14—C13—H13	114.7	O4—C12—H12C	109.5
C11—O4—C12	115.85 (19)	H12A—C12—H12C	109.5
C17—C16—C15	120.5 (2)	H12B—C12—H12C	109.5
C17—C16—H16	119.7	O5—C20—H20A	109.5
C15—C16—H16	119.7	O5—C20—H20B	109.5
C14—C15—C16	120.69 (19)	H20A—C20—H20B	109.5
C14—C15—H15	119.7	O5—C20—H20C	109.5
C16—C15—H15	119.7	H20A—C20—H20C	109.5
C13—C10—C11	119.64 (19)	H20B—C20—H20C	109.5
C13—C10—C9	124.47 (19)	C4—C3—C2	117.0 (3)
C11—C10—C9	115.55 (17)	C4—C3—H3	121.5
O1—C8—N1	124.68 (19)	C2—C3—H3	121.5
O1—C8—C7	129.4 (2)	C7—C6—C5	116.3 (3)
N1—C8—C7	105.93 (18)	C7—C6—H6	121.8
O5—C17—C16	124.9 (2)	C5—C6—H6	121.8
O5—C17—C18	115.70 (19)	O6—C21—H21A	109.5
C16—C17—C18	119.35 (19)	O6—C21—H21B	109.5
N1—C9—C10	113.80 (17)	H21A—C21—H21B	109.5
N1—C9—H9A	108.8	O6—C21—H21C	109.5
C10—C9—H9A	108.8	H21A—C21—H21C	109.5
N1—C9—H9B	108.8	H21B—C21—H21C	109.5
C10—C9—H9B	108.8	C5—C4—C3	121.3 (3)
H9A—C9—H9B	107.7	C5—C4—H4	119.4
C18—C19—C14	121.0 (2)	C3—C4—H4	119.4
C18—C19—H19	119.5	C4—C5—C6	121.9 (3)
C14—C19—H19	119.5	C4—C5—H5	119.0
O6—C18—C19	124.6 (2)	C6—C5—H5	119.0
O6—C18—C17	115.68 (19)		
			
C15—C14—C13—C10	47.1 (3)	C14—C19—C18—O6	−176.9 (2)
C19—C14—C13—C10	−137.5 (2)	C14—C19—C18—C17	4.3 (3)
O3—C11—O4—C12	3.1 (3)	O5—C17—C18—O6	−2.9 (3)
C10—C11—O4—C12	−177.31 (19)	C16—C17—C18—O6	177.6 (2)
C19—C14—C15—C16	−0.7 (3)	O5—C17—C18—C19	176.0 (2)
C13—C14—C15—C16	174.6 (2)	C16—C17—C18—C19	−3.5 (3)
C17—C16—C15—C14	1.4 (4)	C8—N1—C1—O2	178.5 (2)
C14—C13—C10—C11	−179.2 (2)	C9—N1—C1—O2	−5.3 (4)
C14—C13—C10—C9	7.8 (4)	C8—N1—C1—C2	−1.1 (2)
O3—C11—C10—C13	178.0 (2)	C9—N1—C1—C2	175.14 (19)
O4—C11—C10—C13	−1.6 (3)	O1—C8—C7—C2	178.8 (2)
O3—C11—C10—C9	−8.4 (3)	N1—C8—C7—C2	−1.7 (3)
O4—C11—C10—C9	172.04 (17)	O1—C8—C7—C6	−1.6 (4)
C1—N1—C8—O1	−178.7 (2)	N1—C8—C7—C6	177.9 (3)
C9—N1—C8—O1	5.1 (4)	C6—C7—C2—C3	0.3 (4)
C1—N1—C8—C7	1.7 (2)	C8—C7—C2—C3	180.0 (2)
C9—N1—C8—C7	−174.50 (19)	C6—C7—C2—C1	−178.6 (2)
C20—O5—C17—C16	4.3 (4)	C8—C7—C2—C1	1.0 (3)
C20—O5—C17—C18	−175.2 (2)	O2—C1—C2—C7	−179.6 (3)
C15—C16—C17—O5	−178.8 (2)	N1—C1—C2—C7	0.0 (3)
C15—C16—C17—C18	0.7 (4)	O2—C1—C2—C3	1.6 (5)
C8—N1—C9—C10	66.3 (3)	N1—C1—C2—C3	−178.8 (3)
C1—N1—C9—C10	−109.5 (2)	C7—C2—C3—C4	−0.1 (4)
C13—C10—C9—N1	−132.6 (2)	C1—C2—C3—C4	178.6 (3)
C11—C10—C9—N1	54.2 (2)	C2—C7—C6—C5	−0.1 (4)
C15—C14—C19—C18	−2.2 (3)	C8—C7—C6—C5	−179.6 (3)
C13—C14—C19—C18	−177.8 (2)	C2—C3—C4—C5	−0.4 (5)
C21—O6—C18—C19	3.7 (4)	C3—C4—C5—C6	0.7 (6)
C21—O6—C18—C17	−177.4 (3)	C7—C6—C5—C4	−0.4 (5)

### Hydrogen-bond geometry (Å, º)

**Table:**

*D*—H···*A*	*D*—H	H···*A*	*D*···*A*	*D*—H···*A*
C15—H15···O1	0.93	2.55	3.440 (3)	160
C4—H4···O5^i^	0.93	2.50	3.354 (3)	153
C4—H4···O6^i^	0.93	2.58	3.320 (4)	137

Symmetry code: (i) *x*+1, *y*, *z*.
